# Balancing oncological control and immune preservation in the immunotherapy era: revisiting lymph node dissection in non-small cell lung cancer

**DOI:** 10.3389/fonc.2025.1652551

**Published:** 2025-10-15

**Authors:** Tao Jing, Jianbao Yang, Xiaoping Wei, Cheng Wang, Bin Li

**Affiliations:** Department of Thoracic Surgery, The Second Hospital & Clinical Medical School, Lanzhou University, Lanzhou, China

**Keywords:** non-small cell lung cancer, tumor-draining lymph nodes, neoadjuvant therapy, immunotherapy, lymphadenectomy

## Abstract

Systematic lymph node dissection (SLND) has long been widely accepted and established as a standard surgical procedure for lung cancer. In recent years, with the increased detection rate of early-stage non-small cell lung cancer (NSCLC) and the advancement of minimally invasive surgery and enhanced recovery concepts, approaches to lymph node dissection have undergone a notable shift. Previous studies have indicated that extensive removal of non-metastatic lymph nodes may offer uncertain clinical benefits. As a result, alternative strategies such as lobe-specific lymph node dissection (L-SLND) and lymph node sampling have gained attention among thoracic surgeons. In recent years, neoadjuvant chemoimmunotherapy for NSCLC has achieved remarkable success, with tumor-draining lymph nodes (TdLNs) playing a pivotal role in the efficacy of immunotherapy. Lymph node preservation strategies may synergize with immunotherapy by maintaining systemic immune surveillance. Conversely, the removal of non-metastatic lymph nodes could disrupt systemic immunity and exert secondary effects on primary tumors or potential micrometastases. This review summarizes the evolution of lymph node dissection strategies in lung cancer surgery and, in the context of encouraging outcomes with immunotherapy, provides new perspectives on future directions for balancing oncological control with immune preservation.

## Introduction

Non-small cell lung cancer continues to rank among the most significant contributors to cancer mortality worldwide. Comprehensive lymph node dissection (LND) remains a cornerstone for accurate staging, and its oncological benefits in terms of long-term survival outcomes remain controversial. The advent of neoadjuvant chemoimmunotherapy has revolutionized the NSCLC treatment paradigm, highlighting the dual role of TdLNs in staging accuracy and anti-tumor immunity. Traditional approaches advocating extensive LND may inadvertently compromise the immune microenvironment by disrupting TdLN-mediated immune responses, highlighting the urgent need to reevaluate these surgical standards. As our understanding of the immunobiology of TdLNs deepens, it can be reasoned that selectively preserving non-metastatic TdLNs may be necessary to harmonize surgical oncological outcomes with immune function in the immunotherapy era. This review outlines the historical shifts in lymph node dissection strategies, synthesizes immunotherapy-related preclinical and clinical evidence, and offers thoughtful perspectives to guide future investigations into non-systematic lymphadenectomy in this evolving therapeutic landscape.

## Current status of lymph node dissection

Lung cancer is the leading cause of cancer-related mortality worldwide (1). The current guidelines for LND in patients with NSCLC recommend systematic lymph node dissection or lymph node sampling (2). For early-stage NSCLC, including T1, N0, and patients with T1, N1 or T2-3, N0–1 disease who have undergone rigorous preoperative mediastinal lymph node assessment (including mediastinoscopy, mediastinotomy, EBUS, EUS, and CT-guided biopsy), the NCCN guidelines recommend mediastinal lymph node dissection or systematic lymph node sampling as a routine component of lung cancer resections. Specifically, a minimum of one N1 and three N2 stations should be sampled or complete lymph node dissection should be performed. For patients undergoing resection for stage IIIA (N2) disease, formal ipsilateral mediastinal lymph node dissection is indicated. The rationale behind these recommendations lies in the need for accurate staging to guide treatment decisions. This surgical standard has been established for decades, with the primary goal of LND being to reduce the risk of understaging rather than to achieve local tumor control. Notably, the ACOSOG Z0030 trial (3) demonstrated no significant survival difference between systematic mediastinal lymph node dissection (MLND) and systematic mediastinal lymph node sampling (MLNS) in T1–2 N0 patients with rigorous preoperative mediastinal lymph node assessment (8.5 years vs 8.1 years, P = 0.25), although operative mortality and complications were higher in the lymph node sampling group (2.0% vs 0.76%, respectively). Additionally, lymph node dissection required a longer median operative time and greater total chest tube drainage compared to lymph node sampling (15 minutes and 121 mL, respectively). Extensive lymph node dissection improves staging accuracy, enabling the identification of patients with more advanced disease who might otherwise be misclassified, a phenomenon referred to as the Will-Rogers phenomenon or stage migration (4).

Inadequate lymph node sampling can leave the true N stage undetected, leading to a false understaging. Although LND contributes to local cancer control, it has not been shown to improve overall survival in patients with distant metastases. For those without lymph node involvement, LND serves only to confirm a pathological N0 status and does not influence the survival outcomes. Therefore, the oncological benefits of LND are likely limited to patients with resectable pN2 NSCLC who do not have distant micrometastases (5).

According to the lymph node metastasis map published by the International Association for the Study of Lung Cancer (IASLC), the metastatic patterns of NSCLC exhibit lobe-specific characteristics (6). For tumors located in different lung lobes, metastases typically involve specific nodal stations: stations 2, 3, and 4 in the right upper lobe; stations 7, 8, and 9 in the right lower lobe; stations 4, 5, and 6 in the left upper lobe; and stations 7, 8, and 9 in the left lower lobe. Tumor location within the lung lobe independently predicts mediastinal lymph node involvement in specific nodal regions (7). Lobe-specific lymph node dissection (L-SLND) may be a potential surgical approach for early-stage NSCLC.

SLND minimizes the risk of incomplete resection to the greatest extent and provides the most comprehensive N staging. However, with the progress of preoperative examinations and a better understanding of tumor biology, the role of SLND in N staging has been questioned and requires further validation through clinical trials. Surgeons are seeking a more precise, cost - effective, and personalized LN resection strategy. Nevertheless, the survival benefits of SLND still need to be further confirmed.

### Lobe-specific lymph node dissection

Lobe-specific lymph node dissection was initially proposed by Nohl in 1956 as a method for refining lymph node dissection in lung cancer surgery (8). Over the past three decades, numerous retrospective studies have provided evidence for the distinct metastatic patterns of lobe-specific lymph nodes in non-small cell lung cancer (NSCLC), further validating the potential of L-SLND as a more targeted surgical approach (9–12). Notably, Jiang et al. synthesized clinical data from Shanghai, refining the strategies for L-SLND based on comprehensive insights into anatomical considerations and primary tumor location (13, 14). In a pivotal prospective clinical trial, Zhang et al. demonstrated that L-SLND tailored to the patterns of mediastinal lymph node metastasis in cT1N0 invasive NSCLC significantly improved the precision of lymph node dissection, thus enhancing patient outcomes and providing critical insights for L-SLND in clinical practice (14). Specifically, the authors have prospectively defined two criteria for the exclusion of lymph node dissection and four criteria for L-SLND. In patients with tumors located in the apical segments, or those with upper lobe tumors that have negative hilar nodes and no visceral pleural invasion, the lymph nodes in the inferior mediastinum (stations 7, 8, and 9) are preserved. Similarly, in patients with left superior segment tumors and negative hilar nodes, dissection of the 4L lymph nodes is not required, while in patients with left basal segment tumors and negative hilar nodes, lymph node dissection is restricted to stations 4, 5, and 6 (14). This approach ensures that only the lymph nodes directly involved in tumor drainage are removed, while preserving those that are unlikely to harbor metastatic disease, thus aligning with the principles of lobe-specific lymph node dissection.

A meta-analysis (15) comparing SLND with L-SLND revealed that SLND was associated with a higher incidence of complications, including bleeding (4% vs. 2.8%), bronchial secretions (12.1% vs. 7.7%), chylothorax (1.8% vs. 0.7%), and recurrent laryngeal nerve injury (2.4% vs. 1.1%). Furthermore, a pooled analysis of 13 studies involving 11,522 patients demonstrated that L-SLND was associated with superior overall survival (hazard ratio [HR] 0.80, 95% CI: 0.73-0.87) compared to systematic dissection, with no significant differences in recurrence-free survival (HR 0.96, 95% CI: 0.84-1.09). We conducted a comprehensive review of 12 retrospective clinical studies and 1 randomized controlled trial (RCT), all of which examined the outcomes of SLND versus L-SLND. The findings across these 13 studies consistently demonstrate that there are no statistically significant differences in the 5-year disease-free survival (DFS) rates between the two lymph node dissection strategies. Furthermore, 11 of these studies indicate that the 5-year overall survival (OS) rates also show no significant variation between SLND and L-SLND. The detailed summary of these studies, including data on complications and survival, is presented in **Table 1**. Importantly, L-SLND was linked to a lower incidence of postoperative complications, such as chylothorax (risk ratio [RR] 0.54, 95% CI: 0.35-0.85) and arrhythmias (RR 0.74, 95% CI: 0.57-0.97) (16). These findings underscore that, for early-stage NSCLC, L-SLND is increasingly favored by surgeons owing to its comparable survival outcomes to those achieved with SLND. Several retrospective studies have suggested that removal of additional negative lymph nodes does not confer survival benefits (16–18).

**Table 1:** Comparative outcomes of systematic lymph node dissection (SLND) versus lobe-specific lymph node dissection (L-SLND) in NSCLC patients across retrospective and prospective studies.

Author	Year	Country	Study design	Number		Staging (eighth)^c^	5-year OS rate			5-year RFS rate			Early mortality		Postoperative pneumonia	Chylothorax	Arrhythmia
				SLND	L-SLND		SLND	L-SLND	P-value	SLND	L-SLND	P-value	SLND	L-SLND	SLND vs L-SLND	SLND vs L-SLND	SLND vs L-SLND
Kuroda (24)	2021	Japan	Retrospective	265	534	cIA-IIIB	60.2	69	0.09^a^	35.6	44	0.11^a^					
Handa (25)	2021	Japan	Retrospective	128	247	cIB-IIIA cT2-3N0-1M0	81.6	75.5	0.17	57.2	58.5	0.53					
Hattori (26)	2021	Japan	Retrospective	181	278	cIA-IB cT1/2aN0M0	78.8	79.9	0.665	70.4	66.5	0.669	90 d 1/181	90 d 3/278	21.0%vs14.7%	7.7%vs1.4%	11.0%vs10.1%
Zhao (27)	2021	China	Retrospective	446	100	cIA cT1a-cN0M0	92	96.7	0.411	88.8	95.6	0.13					3%vs1%
Wang (28)	2019	China	Retrospective	328	577	pT1a-2aN0M0	80	77.5	>0.05	75	70.5	>0.05					
Adachi (29)	2017	Japan	Retrospective	190	145	cT1-3N0-1M0	75.3	73.5	0.977^a^								
Hishida (30)	2016	Japan	Retrospective	4124	1268	cIA-IIIA/cT1-4N0-1M0	75.9	81.5	<0.001				19/4124	5/1268	1.9%vs1.2%	1.3%vs1.0%	3.1%vs2.5%
Shapiro (31)	2013	USA	Retrospective	282	88	cIA-IIIA	82	89	0.36	68	74	0.12	2/282	0/88			
Ma (32)	2013	China	RCT	51	45	cIA-IB/cT1a-bN0M0	64.9	69.7	0.552	60.8	66	0.241			9.8%vs4.4%	3.9%vs0	3.9%vs2.2%
Maniwa (33)	2013	Japan	Retrospective	206	129	cIA-IIIA	89.7	86.6	0.526	77.7	76.4	0.607	0/206	0/129	1.5%2.3%	2.9%vs2.3%	9.7%vs4.7%
Jiang (34)	2013	China	Retrospective	309	94	cIA-IIA	74.6	68.5	0.216				0/309	0/94	4.5%vs7.4%^b^		
Ishiguro (35)	2010	Japan	Retrospective	625	147	cIA-IIIC	71.9	76	0.29								
Okada (36)	2006	Japan	Retrospective	358	377	cIA-IIIA	79.7	83.2	0.06	73.4	76.4	0.376			4.2%vs1.6%	1.1%vs0.5%	5.3%vs3.2%

^a^The results of propensity-score matched comparison.

^b^Including all postoperative morbidity.

^c^Re-iterated based on the eighth TNM staging.

SLND, Systematic Lymph Node Dissection; L-SLND, Lobe-Specific Lymph Node Dissection; OS, Overall Survival; RFS, Recurrence-Free Survival; RCT, Randomized Controlled Trial

Distinct mediastinal lymph node metastasis patterns are observed in early-stage NSCLC. Based on the unique characteristics of lobe-specific lymphatic drainage, L-SLND maybe a promising alternative to systematic lymph node dissection in the treatment of select patients with early-stage NSCLC (13, 16). The ongoing Japanese Clinical Oncology Group (JCOG) clinical trial (JCOG1413) is currently investigating the clinical benefits of L-SLND, and the results may provide further evidence supporting the preservation of lymph node dissection in these patients (19, 20). While previous studies have primarily focused on early-stage lung cancer, more recent analyses of survival data from N1 patients undergoing L-SLND have indicated that following propensity score matching, the rate of N2 lymph node metastasis was higher in the systematic lymph node dissection group (55.4% vs. 41%, P = 0.087). However, no significant differences were observed in the total recurrence rates (48.2% vs. 54.2%, P = 0.60) or lymph node recurrence rates (14.5% vs. 20.5%, P = 0.41) between the two groups (21). These findings suggest that in N1 NSCLC, the primary role of lymph node dissection may be to prevent understaging rather than to improve prognosis.

A retrospective study conducted by Deng et al (22) further explored the impact of lymph node dissection on the efficacy of immunotherapy in patients with NSCLC with tumor recurrence. Their findings indicated that the greater the number of dissected lymph nodes (particularly those exceeding 16), the poorer the efficacy of subsequent immunotherapy. This observation challenges the “more is better” philosophy and suggests that a precise, rather than extensive, lymph node dissection strategy is preferable to preserve the integrity of immunologically important non-lobe-specific lymph nodes. This insight has given rise to the concept of immune-driven lymph node dissection strategies. In a melanoma mouse model (23), resident memory T cells (T_RM_), which are abundant in lymph nodes and are crucial for long-term tumor immunity, could be induced through the loss of regulatory T cells (Tregs) during the neoadjuvant phase. These T_RM_ cells may play a pivotal role in restricting tumor metastasis. Although the ability of immune checkpoint inhibitor (ICI) treatment to mobilize T_RM_ cells from the lymph nodes remains uncertain, it is hypothesized that ICI treatment may activate these immune populations, providing a potential explanation for the better immunotherapeutic responses observed in patients who undergo more limited lymph node dissection. These findings suggest that lobe-specific lymph node dissection (L-SLND) could be a promising alternative for carefully selected early-stage NSCLC patients. Nevertheless, its oncological safety and immunological benefits still need to be confirmed by robust prospective clinical trials and experimental studies in order to validate these hypotheses and establish evidence-based, immune-driven lymph node dissection strategies.

### The dawn of neoadjuvant chemotherapy-immunotherapy

Recent advancements in neoadjuvant chemotherapy-immunotherapy combinations have marked a transformative shift in the treatment of resectable NSCLC. The establishment of the perioperative concept for NSCLC, introduced in 2023, represents a landmark development in thoracic oncology (37). This progress has been significantly accelerated by the positive outcomes of four major randomized phase-III trials published in 2024. Among these, the Keynote-671 trial, a randomized, double-blind phase III study presented at American Society of Clinical Oncology (ASCO) 2023, evaluated the efficacy of perioperative pembrolizumab in patients with NSCLC. The results demonstrated a two-year overall survival (OS) rate of 80.9% for pembrolizumab compared to 77.6% for placebo (P = 0.02), along with a major pathological response (MPR) rate of 30.2% versus 11.0% (P<0.0001) and a pathological complete response (pCR) rate of 18.1% versus 4.0%, respectively(P<0.0001) (38).

Similarly, other pivotal trials, including the AEGEAN trial (presented at AACR 2023) (39), Checkmate-77T (presented at European Society for Medical Oncology 2023) (40), and Neotorch trial (presented at the ASCO Virtual Plenary, April 2023) (41), reported comparable survival outcomes, further reinforcing the efficacy of combining immune checkpoint inhibitors with chemotherapy in the perioperative setting. Taken together, these studies suggest that the combination of immunotherapy and chemotherapy during the perioperative period provides superior outcomes compared with chemotherapy alone in patients with resectable NSCLC.

Across the pivotal KEYNOTE-671, AEGEAN, and CheckMate 77T trials, a consistent pattern emerged in which patients with stage III disease demonstrated lower hazard ratios for event-free survival (EFS) compared with those with stage II disease, suggesting enhanced benefit from chemoimmunotherapy in more advanced stages; notably, in CheckMate 77T, the subgroup with multi-station N2 involvement achieved the lowest HR, underscoring that the addition of immunotherapy to chemotherapy confers superior EFS even in patients with extensive nodal disease, although it should be emphasized that statistical significance (P-values) was not reported for these subgroup analyses (**Table 2**). Complementing these trial data, a pooled analysis by Zhai et al. further demonstrated that neoadjuvant immunotherapy exerts pronounced efficacy in patients with metastatic lymph nodes, supporting the hypothesis that preoperative chemoimmunotherapy may be particularly advantageous for individuals with nodal metastasis, especially those with cN2 disease.

**Table 2:** The hazard ratio for event-free survival (EFS) under different clinical stages and lymph node stages.

Study	Clinical disease stage			Lymph node station
	II	IIIA	IIIB	
KEYNOTE-671 (38)	0.76 (0.43–1.34)	0.57 (0.39–0.83)	0.83 (0.52–1.32)	N2 single:0.61 (0.39–0.94) N2 multi:0.69 (0.33–1.38)
AEGEAN (39)	0.65(0.42-1.01)	0.54(0.42-0.7)		
CheckMate 77T (40)	0.81 (0.46–1.43)	0.51 (0.36–0.72)		N0:0.51 (0.36–0.72) N1:0.58 (0.29–1.16) N2 single:0.49 (0.29–0.84) N2 multi:0.43 (0.21–0.88)

Notably, the favorable results observed in these trials can be attributed to the strategic sequencing of treatments, where immunotherapy is administered prior to surgery. This sequencing is critical because surgery and radiotherapy can impair TdLN-mediated immunity, potentially compromising subsequent treatment efficacy. However, there are currently no prospective studies investigating different lymph node dissection approaches in the setting of perioperative immunotherapy, and caution should be exercised when making inferences that go beyond the existing evidence.

### Tumor-draining lymph nodes and tumor immunobiology

**Figure 1:**
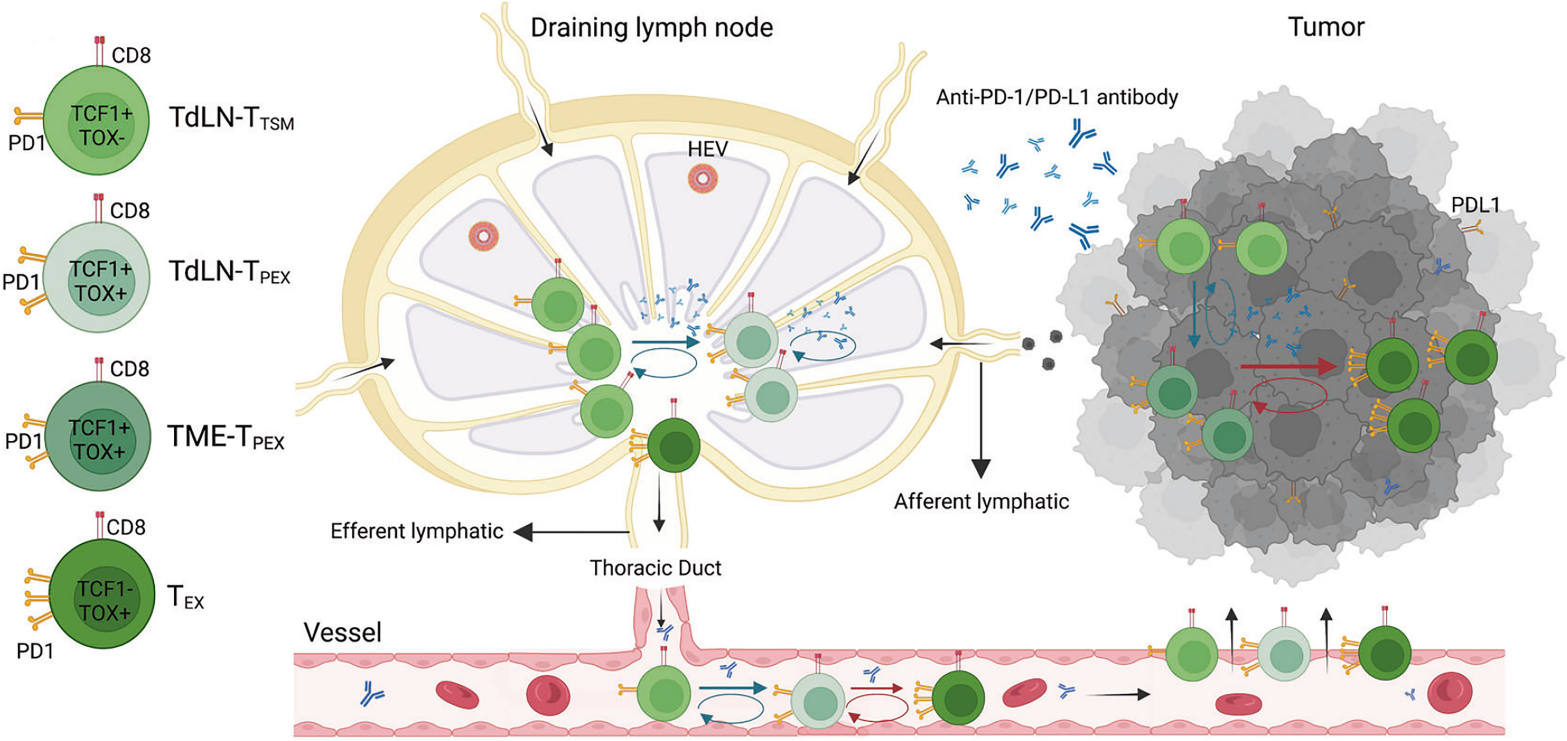
The mechanism of tumor draining lymph nodes-tumor specific memory T cells (TdLN-TTSM) response to PD-1/PD-L1 immune checkpoint blocking. As a precursor of TdLN-progenitor of exhausted T cells (TPEX), TTSM is located upstream of differentiation and persistently recruit various exhausted T cell subpopulation located in the tumor microenvironment (TME). The antitumor effect of PD-1/PD-L1 antibody is dependent on TTSM cell subsets. The prerequisite for antitumor effect of PD-1/PD-L1 antibody is to first amplify TTSM cell in draining lymph nodes, meanwhile promoting the differentiation into TPEX which subsequently differentiate into exhausted CD8+ T cells (TEX). Finally, the progeny of these cells enters TME through peripheral circulation and plays an antitumor role (43).

Tumor-draining lymph nodes situated along the lymphatic drainage pathways of primary tumors serve as critical sites for the activation of anti-tumor lymphocytes through the presentation of tumor-specific antigens. These lymph nodes act as reservoirs for tumor-specific T cells, playing a central role in the initiation of tumor antigen recognition and subsequent activation of anti-tumor immune responses. Compared with normal lymph nodes, TdLNs are characterized by a higher number of PD-L1^+^ immune cells, including macrophages, migratory conventional dendritic cells (cDCs), cDC2s, and resident CD8α^+^ dendritic cells, which contribute to the immune milieu (42). Additionally, TCF-1^+^TOX^+^CD8^+^ T cells(TCF-1: T-cell Factor 1) and tumor-specific memory T cells derived from TdLNs have been identified as primary responders to PD-1/PD-L1 blockade therapy, highlighting their pivotal role in enhancing immunotherapy efficacy (43, 44) (**Figure 1**).

Selective irradiation of TdLNs that have not undergone metastasis can disrupt the chemokine-driven recruitment of effector T cells, thereby reducing the effectiveness of combined radiotherapy and immunotherapy approaches (45). Furthermore, extensive dissection of TdLNs may result in immune dysfunction, as these lymph nodes are essential for tumor antigen presentation and subsequent antigen-specific immune activation (46–48). Tumor-specific memory CD8+ T cells originating from TdLNs are critical for the efficacy of PD-1 blockade therapy, and their removal through surgical dissection can prevent tumor regression induced by immunotherapy. This disruption is associated with a reduction in immune cell infiltration within the tumor microenvironment (43). Experimental studies on immune checkpoint blockade combined with selective dissection of TdLNs have underscored the indispensable role of these lymph nodes in mediating the immune response to cancer immunotherapy (45, 49).

During clinical antitumor therapy, dendritic cells capture antigens released from dying tumor cells and subsequently migrate to tumor-draining lymph nodes, where they initiate the priming of naïve T cells. These naïve T cells then proliferate and differentiate into effector and memory T cells. Activated effector T cells exit the lymph nodes, infiltrate tumor sites, and specifically recognize and eliminate malignant cells while releasing additional tumor antigens that further fuel immune activation. In parallel, memory T cells can persist long-term within the host, rapidly expanding and differentiating upon re-encounter with the same antigen, thereby sustaining antitumor immunity through a positive feedback loop of immune checkpoint inhibition (50, 51). Thus, tumor-draining lymph nodes function as both “training centers” for effector T-cell development and hubs of innate–adaptive immune crosstalk that critically shape therapeutic outcomes. Importantly, clinical evidence supports (52) that immune checkpoint inhibitors (ICIs) can induce the priming and systemic trafficking of CD8+ T cells from extracranial tumors, conferring not only local control but also robust efficacy against distant metastatic lesions, such as the intracranial responses observed in melanoma brain metastases. These findings suggest that preserved lymph nodes remain immunologically relevant even in the context of distant metastases, as they continue to serve as key sites for T-cell activation and systemic immune surveillance during immunotherapy.

In light of these compelling findings and consistent with recent data from Deng (22), we hypothesized a significant correlation between lymph node dissection strategies and the effectiveness of immunotherapy. This hypothesis is further supported by preclinical research that emphasizes the critical role of TdLNs in facilitating immune responses that enhance the success of immunotherapeutic interventions.

## Preclinical studies on tumor-draining lymph nodes

In a notable study by Fear (53), a murine model demonstrated that complete lymph node excision resulted in significantly reduced survival rates in tumor-bearing mice. In contrast, partial lymph node excision or early administration of aPD-1/aCD40 therapy improved survival outcomes. Data from a lung metastasis mouse model revealed that excision of the primary subcutaneous tumor in combination with varying extents of drainage lymph node (dLN) removal significantly influenced survival. The group that underwent complete lymph node excision exhibited a markedly shorter median survival time (49 days) than the intact lymph node group (88 days; P < 0.05). The partial lymph node excision group demonstrated partial recovery in survival, which was dependent on the presence of CD8+ T cells. These findings align with similar observations in the studies by Fransen (54), emphasizing the importance of lymph node preservation in enhancing immune-mediated tumor responses.

A population of tumor-specific CD8+ T cells exists within TdLNs, exhibiting memory characteristics without functional exhaustion. These TdLN-derived tumor-specific memory (T_TSM_) cells establish early epigenetic programs linked to immune memory during the initial stages of tumorigenesis. Importantly, T_TSM_ cells from TdLNs show enhanced anti-tumor treatment efficacy following adoptive transfer and are recognized as primary responders to PD-1/PD-L1 blockade therapy (44, 55). In murine models using a colon adenocarcinoma cell line with both unilateral and bilateral subcutaneous xenografts, bilateral excision of TdLNs, rather than unilateral excision, significantly reduced the anti-tumor effects and *in vitro* efficacy of combined immunotherapy-radiation therapy (iRT). TdLNs play a crucial role in promoting CD8+ T cell infiltration and sustaining M1/M2 macrophage populations in the iRT paradigm (45). These findings underscore the importance of preserving normal non-metastatic TdLNs in the context of immunotherapy, as their retention may confer therapeutic advantages over traditional chemotherapy approaches. Nevertheless, evidence from animal models is still scarce and generally regarded as low-level. Findings from preclinical studies should not be hastily or uncritically extrapolated to clinical practice. Consequently, there is a clear need for large-scale, rigorously designed preclinical investigations to further test and substantiate this hypothesis.

## Exploration of TdLNs in immunotherapy for other cancer types

The critical immunological functions of TdLNs challenge the traditional view that PD-1/PD-L1 checkpoint inhibitors predominantly exert their effects at tumor sites. In patients with microsatellite instability-high (MSI-H) or mismatch repair-deficient (dMMR) colorectal cancer, extensive lymph node dissection has shown minimal clinical benefits in terms of long-term survival outcomes (56). A Phase I clinical trial in oral squamous cell carcinoma demonstrated that stereotactic radiotherapy targeting tumor lesions, while sparing uninvolved TdLNs, significantly increased the rate of pathological complete response (57). Conversely, two Phase III trials (58, 59) involving head and neck squamous cell carcinoma, which combined standard lymph node radiotherapy with immunotherapy, failed to show significant improvements in overall survival (OS) or event-free survival (EFS).

These findings suggest that immunotherapy, when combined with standard tumor treatments, may require a carefully optimized sequencing approach to effectively activate immune surveillance and modulate primary tumor responses. Specifically, initiating immunotherapy before lymph node-targeted treatment, even in cases involving metastatic nodes, could be pivotal for maximizing therapeutic outcomes. Conventional oncological strategies targeting TdLNs may inadvertently impair host anti-tumor immunity by excising critical secondary lymphoid organs, thus compromising the immune response to immunotherapy (60). In the context of PD-1/PD-L1 blockade therapy, TdLNs are integral in generating a substantial population of fully differentiated anti-tumor T cells, which are essential for effective tumor control (47, 61). These T cells play a significant role in regulating immune responses and facilitating tumor regression (62, 63). Collectively, these findings highlight that an intact nodal basin may offer potential therapeutic benefits by supporting systemic immune surveillance, and lymph node preservation strategies could serve as an immunological basis for effective immunotherapy. Nevertheless, the supporting evidence primarily comes from retrospective studies with small cohorts, which inherently carry a lower level of evidence. Thus, prospective, rigorously designed large-sample studies are warranted to confirm these hypotheses.

## Preoperative lymph node evaluation

The key premise for adopting selective lymph node dissection strategies is the accurate determination of lymph node involvement by tumor cells, enabling the precise excision of negative lymph nodes. 18F-FDG PET-CT, a widely used imaging technique that reflects glucose metabolism, plays a central role in the diagnosis of malignant tumors. For patients preoperatively diagnosed with cT1-2N0M0 and for those planned for immunotherapy, strategies such as lobectomy-specific lymph node dissection or lymph node sampling—tailored to the primary tumor’s location—are employed. This approach enhances subsequent immunotherapy efficacy by optimizing immune surveillance within the lymph nodes. However, a significant discrepancy was observed in the response of TdLNs compared with the primary tumor site following neoadjuvant immunotherapy. Specifically, tumor-infiltrating positive lymph nodes did not exhibit a reduction in metabolic activity, whereas tumor-negative lymph nodes showed a marked decrease. In patients achieving pathological complete response (pCR) or major pathological response (mPR), TdLNs metabolic activity significantly increases after immunotherapy, a phenomenon unique to immunotherapy as opposed to chemotherapy (64, 65). Studies have shown that the highest glucose uptake within tumors occurs in bone marrow cells, followed by T cells and cancer cells, with the latter primarily utilizing glutamine and lipids for metabolism (66). Thus, post-immunotherapy PET-CT may be insufficient for assessing mediastinal lymph node involvement, suggesting the need for more accurate preoperative or intraoperative lymph node evaluation despite the practical challenges involved. Currently, no imaging or invasive techniques reliably identify metastatic lymph nodes, although future predictive models leveraging machine learning, artificial intelligence (67), or glutamine-based PET-CT imaging (65) may offer more reliable solutions. These advancements could form the foundation for less invasive and selective lymph node dissection strategies.

Over the past four decades, minimally invasive techniques have revolutionized thoracic surgery. However, focusing exclusively on minimizing the incision size and number has proven suboptimal. In response, the concept of “Minimally Invasive Surgery 3.0” (MIS 3.0) has emerged, which emphasizes reducing the resection scope while minimizing systemic trauma (68). This shift reflects the growing need for more precise and personalized approaches for lung cancer resection. We cautiously advocate performing appropriate, rather than excessive, lymph node dissection—accurately excising all metastatic nodes while preserving uninvolved nodes. Although systematic lymph node dissection remains vital for precise cancer staging, future considerations should prioritize avoiding unnecessary lymph node resection in patients with no evidence of metastasis or those showing favorable responses to immune checkpoint blockade (ICBs) (69). To improve the preoperative estimation of lymph node dissection requirements, techniques such as endobronchial ultrasound (EBUS)-guided biopsy, as well as assessments of metastatic status, differentiation, molecular markers, immune microenvironment, and memory cell content, should be considered to evaluate tumor invasiveness. Furthermore, the use of PET/CT to assess SUVmax values (70) and lymph node size may assist in identifying potential metastatic lymph node locations and estimating metastatic burden (71). These methods provide promising avenues for enhancing preoperative assessment, enabling surgeons to tailor lymph node dissection with greater precision. The benefits of lymph node preservation are particularly significant for enhancing immunotherapy outcomes, with a relatively minimal impact on chemotherapy efficacy. This may explain the historical emphasis on extensive lymph node excision prior to immunotherapy (21).

## Potential risks and limitations of SLND

Despite its theoretical immunological advantages, SLND may pose significant oncological risks. For instance, under-staging or missing occult N2 metastasis could negatively impact patient outcomes. Evidence from the ACOSOG Z0030 trial demonstrated that comprehensive mediastinal lymph node dissection (MLND) still detects a notable proportion of unexpected N2 disease (4%). Robinson (72) and Nitadori’s studies (73) have demonstrated that occult N1 nodal metastases are common in patients with clinical N0 peripheral, small (<=2 cm) NSCLC. These metastases are often found in more peripheral interlobar, lobar, and segmental stations, suggesting that the distribution of occult metastases varies based on tumor location. Moreover, histological subtypes play a significant role in the risk of occult metastasis. Specifically, the micropapillary subtype has been identified as an independent predictor of occult N2 mediastinal lymph node metastasis. Furthermore, research by Chen (74) and Shin (75) indicates that centrally located lung cancers are one of the key predictors of occult N2 metastasis. This finding underscores the importance of considering tumor location when assessing the risk of occult N2 involvement in NSCLC patients. Therefore, careful patient selection, high-quality preoperative imaging, intraoperative frozen section assessment, and thorough surgical exploration remain indispensable when considering limited dissection strategies, especially in patients with higher-stage tumors or ambiguous nodal status. Further prospective studies are required to better define the safety margins of SLND in the setting of neoadjuvant immunotherapy.

Due to limited understanding of lymphatic drainage patterns, incomplete analysis of all relevant clinical characteristics, and variations in surgical strategies across different regions, lymph node dissection strategies remain inadequately defined. Each innovative approach requires further supporting evidence from studies conducted in diverse clinical contexts. Additionally, while lymph nodes play a critical role in immune surveillance, their removal may potentially impact immune homeostasis, influencing the incidence or severity of immunotherapy-related adverse events, such as immune-related pneumonia or myocarditis. However, this relationship remains largely unexplored and warrants further investigation. Looking ahead, with the advancement of translational medicine, the continuous expansion of clinical datasets, and the increasing analytical power of new tools, more precise and individualized lymph node dissection strategies can be anticipated in the future.

Lymph nodes are not merely passive conduits of tumor spread but serve as active microenvironments that influence the course of disease. They function as reservoirs for micrometastases and dormant tumor cells, hubs of immunosuppression through regulatory immune cell expansion, and sites of stromal and extracellular matrix remodeling that foster pre-metastatic niche formation. Even when macroscopically normal, a significant proportion of lymph nodes may harbor occult disease, enabling locoregional recurrence or systemic dissemination if left behind after primary tumor resection (76, 77). Conversely, systematic lymph node dissection reduces tumor burden and eliminates potential sources of recurrence, although it cannot fully prevent progression once micrometastases have seeded distant organs. This dual role of lymph nodes—both as facilitators of metastasis and as sites of immune regulation—explains why their involvement critically shapes oncological outcomes and underscores the rationale for comprehensive lymph node management in lung cancer surgery.

## Conclusion

The favorable outcomes associated with immunotherapy, together with the controversial survival benefit of complete lymph node excision, highlight the need for further careful evaluation rather than an immediate change of lymph node management strategies. The evidence reviewed in this paper underscores the potential immunological significance of tumor-draining lymph nodes, but we acknowledge that this concept remains hypothesis-generating and requires validation in well-designed prospective randomized trials. TdLNs function both as immune barriers and as common sites of metastasis, presenting opportunities and challenges in harnessing their anti-tumor effects while minimizing oncological risks. At present, however, systematic lymph node dissection should remain the standard of care until the results of the ongoing JCOG1413 trial are released and the oncological safety and potential benefits of lobe-specific lymph node dissection are definitively established. Further advancing our understanding of TdLN immunobiology, particularly the protective value of preserving non-metastatic nodes and the evolution of the immune microenvironment during immunotherapy, will be crucial. Integrating emerging data may help inform future clinical practice, requiring close collaboration among basic researchers, thoracic surgeons, oncologists and large-scale AI models to develop evidence-based and feasible lymphadenectomy strategies for the era of immunotherapy.
